# Fostering innovation and advancement in evidence‐based practice and guidelines within the field of pediatrics: The 2024 BRIGHT declaration

**DOI:** 10.1002/pdi3.2513

**Published:** 2024-12-10

**Authors:** Xu Wang, Hui Liu, Janne Estill, Fujian Song, Akihiko Ozaki, Juan V. A. Franco, Ivan D. Florez, Etienne Ngeh, Nav Persaud, Liliya Eugenevna Ziganshina, Myeong Soo Lee, Lu Zhang, Yuan Chi, Yuting Duan, Enmei Liu, Yaolong Chen, Xiaodong Zhao

**Affiliations:** ^1^ National Clinical Research Center for Child Health and Disorders, Ministry of Education Key Laboratory of Child Development and Disorders Chongqing Key Laboratory of Child Infection and Immunity China International Science and Technology Cooperation base of Child Development and Critical Disorders Chongqing China; ^2^ Chevidence Lab Child & Adolescent Health Department of Pediatric Research Institute Children's Hospital of Chongqing Medical University Chongqing China; ^3^ Evidence‐based Medicine Center School of Basic Medical Sciences Lanzhou University Lanzhou Gansu China; ^4^ Institute of Global Health University of Geneva Geneva Switzerland; ^5^ Norwich Medical School University of East Anglia Norwich UK; ^6^ Breast and Thyroid Center Jyoban Hospital of Tokiwa Foundation Fukushima Japan; ^7^ Institute of General Practice Medical Faculty, Heinrich‐Heine‐Universitat Dusseldorf Dusseldorf Nordrhein‐Westfalen Germany; ^8^ Department of Pediatrics University of Antioquia Medellin Colombia; ^9^ School of Rehabilitation Science McMaster University Hamilton Ontario Canada; ^10^ Pediatric Intensive Care Unit Clinica Las Americas‐AUNA Medellin Colombia; ^11^ School of Health and Well Being Sheffield Hallam University Sheffield UK; ^12^ Research Organisation for Health Education and Rehabilitation‐Cameroon (ROHER‐CAM) Bamenda Cameroon; ^13^ Department of Family Medicine University of Toronto Faculty of Medicine Toronto Ontario Canada; ^14^ Russian Medical Academy for Continuing Professional Education (RMANPO) Moscow Russian Federation; ^15^ The Peoples' Friendship University of Russia named after Patrice Lumumba (RUDN University) Moscow Russian Federation; ^16^ The Kazan State Medical University Kazan Russian Federation; ^17^ KM Science Research Division Korea Institute of Oriental Medicine Daejeon Republic of Korea; ^18^ Department of Computer Science Hong Kong Baptist University Hong Kong China; ^19^ Institute of Systems Medicine and Health Sciences Hong Kong Baptist University Hong Kong China; ^20^ Yealth Network Beijing Health Technology Co., Ltd Beijing China; ^21^ The Affiliated Traditional Chinese Medicine Hospital Guangzhou Medical University Guangzhou China; ^22^ Department of Respiratory Medicine Children's Hospital of Chongqing Medical University Chongqing China; ^23^ Research Unit of Evidence‐based Evaluation and Guidelines Chinese Academy of Medical Sciences School of Basic Medical Sciences Lanzhou University Lanzhou China; ^24^ Key Laboratory of Evidence‐based Medicine of Gansu Province Lanzhou China; ^25^ Institute of Health Data Science Lanzhou University Lanzhou China; ^26^ Division of Rheumatology and Immunology Children's Hospital of Chongqing Medical University Chongqing China

**Keywords:** declaration, evidence‐based medicine, guidelines, pediartic

## Abstract

The first Better evidence and RecommendatIons for the next Generation HealTh—BRIGHT symposium was held in Chongqing, China between June 21 and 23, 2024. The symposium did not only showcase the recent progress made by multidisciplinary teams in generating and translating evidence for children's healthcare, guideline development and evaluation, and the utilization of AI applications in pediatrics but also fostered transnational and transregional cooperation to promote the advancement of high‐quality evidence and guidelines in this field. The symposium contributed significantly to the future development and transformation of research endeavors in pediatrics.

The first Better evidence and RecommendatIons for the next Generation HealTh—BRIGHT symposium was held in Chongqing, China between June 21 and 23, 2024. It was organized by the Chevidence Lab for Child and Adolescent Health, Children's Hospital of Chongqing Medical University. It attracted a diverse group of researchers and nationalities from various countries, including China, Cameroon, Canada, Colombia, the United Kingdom, Germany, Japan, South Korea, Russia, and Switzerland, attended the symposium. The aim of BRIGHT symposium was to foster innovation and advancement in evidence‐based practice and guideline development within the field of pediatrics.

The BRIGHT symposium brought together a diverse range of professionals including clinicians, methodologists, epidemiologists, artificial intelligence (AI) experts, policy makers, and journal editors. The symposium did not only showcased the recent progress made by multidisciplinary teams in generating and translating evidence for children's healthcare, guideline development and evaluation, and the utilization of AI applications in pediatrics but also fostered transnational and transregional cooperation to promote the advancement of high‐quality evidence and guidelines in this field. The symposium contributed significantly to the future development and transformation of research endeavors in pediatrics.

The constantly increasing availability of new methods, tools, and resources, particularly those related to AI, not only offer great opportunities to enhance pediatric research and develop high‐quality guidelines but also come with challenges. Only through enhancing international cooperation and exchanges and collectively addressing these challenges can we promote sustainable and robust development of evidence‐based pediatric practice in China and beyond.

## DURING THE BRIGHT SYMPOSIUM, THE PARTICIPANTS ENGAGED IN A CANDID AND DYNAMIC DISCOURSE. THE DISCUSSIONS RESULTED IN THE FORMULATION OF THE BRIGHT DECLARATION CONSISTING OF THE FOLLOWING TEN CALLS FOR ACTION:

1. To standardize the terminology in the field of rare childhood diseases, enhancing the quality of clinical research in this area, and developing evidence‐based guidelines and expert consensus statements for rare childhood diseases.
2. To establish a comprehensive set of core outcome indicators for children and promptly applying it in clinical research, evidence synthesis, and clinical practice guideline.
3. To create a repository for evidence on off‐label treatment use specifically for children to promote scientific and standardized off‐label medication practices among pediatricians and general practitioners.
4. To promote the establishment of nationwide and international registries of pediatric patients with rare diseases to overcome the limitations caused by small sample sizes in clinical studies of rare diseases.
5. To enhance adherence and improve compliance to reporting standards in pediatric research by utilizing AI technology.
6. To enhance the utilization of AI technology to expedite the generation and synthesis of evidence.
7. To harness AI capabilities to accelerate the development, reporting, evaluation, and implementation processes associated with guidance documents.
8. To support the recognition of the significance of high‐quality international journals as platforms for publishing and disseminating pediatric evidence and guidelines.
9. To further enhance and broaden international collaboration to facilitate the dissemination of high‐quality evidence and guidelines among pediatricians as well as children and their families globally.
10. To organize the BRIGHT symposium regularly and establish a renowned regional and global platform for academic discussions related to evidence‐based pediatrics.

## THE FOLLOWING COMMITMENTS AND ACTIONS WILL BE UNDERTAKEN BY ORGANIZERS OF THE BRIGHT SYMPOSIUM:

- To actively translate the outcomes of the conference into practical actions, fostering innovation and advancement in evidence‐based practice and guideline development within pediatrics.
- To enhance collaboration and exchanges with organizations, enterprises, and research institutions globally to collectively advance in‐depth research and application of evidence‐based practices and guidelines in pediatrics.
- To monitor closely the key issues and challenges raised in the first BRIGHT symposium and effective measures to address them.

The organizers extend their sincere gratitude to all participants for their enthusiastic engagement and valuable contributions. With our collective endeavors, we are confident that BRIGHT will yield fruitful outcomes and infuse fresh vitality into the global advancement of pediatric evidence and guidelines. Let us collaborate harmoniously toward a BRIGHTer future!

## ATTENDEES OF BRIGHT SYMPOSIUM (LISTED IN ALPHABETICAL ORDER BY LAST NAME):

Jie Cai (Beijing, China), Yaolong Chen (Lanzhou, China), Yuan Chi (Hamilton, Canada), Yupeng Cun (Chongqing, China), Minjie Duan (Chongqing, China), Yuting Duan (Guangzhou, China), Janne Estill (Geneva, Switzerland), Ivan D. Florez (Medellin, Colombia), Juan VA Franco (Düsseldorf, Germany), Long Ge (Lanzhou, China), Dan Hong (Chengdu, China), Ziyu Hua (Chongqing, China), Honghao Lai (Lanzhou, China), Myeong Soo Lee (Seoul, South Korea), Ruobing Lei (Chongqing, China), Xueping Li (Chongqing, China), Yueyan Li (Chongqing, China), Zeming Li (Hongkong, China), Enmei Liu (Chongqing, China), Hanxiang Liu (Chongqing, China), Xiang Liu (Chongqing, China), Hui Liu (Lanzhou, China), Jiawei Luo (Chengdu, China), Qing Luo (Chongqing, China), Xufei Luo (Lanzhou, China), Han Lv (Beijing, China), Etienne Ngeh (Douala, Cameroon), Akihiko Ozaki (Fukushima, Japan), Nav Persaud (Toronto, Canada), Yishan Qin (Chongqing, China), Fujian Song (Norwich, UK), Jing Sun (Beijing, China), Shilin Tang (Chongqing, China), Fan Wang (Chongqing, China), Xu Wang (Chongqing, China), Zhixiang Wang (Beijing, China), Ximing Xu (Chongqing, China), Shu Yang (Chengdu, China), Xuan Yu (Lanzhou, China), Pengfei Zhang (Chengdu, China), Shu Zhang (Chongqing, China), Jungang Zhao (Chongqing, China), Xiaodong Zhao (Chongqing, China), Yao Zhao (Chongqing, China), Lu Zhang (Hongkong, China), and Liliya Eugenevna Ziganshina (Moscow, Russia).

## CONFLICT OF INTEREST STATEMENT

Yaolong Chen and Xiaodong Zhao are Editors‐in‐Chief of Pediatric Discovery. Enmei Liu, Janne Estill, and Ivan D. Florez are Deputy Editors‐in‐Chief of Pediatric Discovery. They were excluded from the peer review process or any editorial decisions related to this manuscript. Akihiko Ozaki received personal fees from MNES, Kyowa Kirin Inc., Becton, Dickinson and Company, Pfizer, Daiichi Sankyo Inc, and Taiho Pharmaceutical Co., Ltd., outside the scope of the submitted work.

## Data Availability

Research data are not shared.

